# A natural nephroprotective adjuvant for cancer chemotherapy: Rosmarinic acid disrupts IL-17 A-Ferroptosis coupling in Ifosfamide-induced renal injury

**DOI:** 10.1007/s12032-026-03280-z

**Published:** 2026-03-08

**Authors:** Büşra Süzen Celbek, Hasan Şimşek, Nurhan Akaras, Özge Kandemir, Hamit Emre Kızıl, Hüseyin Mutlu, Fatih Mehmet Kandemir

**Affiliations:** 1 Department of Pediatrics, Liv Hospital Ankara, Ankara, Türkiye; 2 Department of Physiology, Faculty of Medicine, Aksaray University, Aksaray, Türkiye; 3 Department of Histology and Embryology, Faculty of Medicine, Aksaray University, Aksaray, Türkiye; 4 Department of Food Processing, Aksaray Technical Sciences Vocational School, Aksaray University, Aksaray, Türkiye; 5 Department of Medical Services and Techniques, Vocational School of Health Services, Bayburt University, Bayburt, Türkiye; 6 Department of Emergency Medicine, Faculty of Medicine, Aksaray University, Aksaray, Türkiye; 7 Department of Medical Biochemistry, Faculty of Medicine, Aksaray University, Aksaray, Türkiye

**Keywords:** Acute kidney injury, Apoptosis, Ferroptosis, Ifosfamide, IL-17A, Inflammation, Nephrotoxicity, Oxidative stress, Rosmarinic acid

## Abstract

Rosmarinic acid (RA) is a natural polyphenol with established pleiotropic protective effects. Ifosfamide (IFA) is a potent antineoplastic agent whose clinical utility is severely limited by dose-dependent nephrotoxicity, primarily mediated by its metabolite, chloroacetaldehyde (CAA). This study aimed to investigate whether RA protects against IFA-induced nephrotoxicity and to elucidate the underlying molecular mechanisms. Male *Wistar albino* rats (*n* = 7 per group) were allocated into four groups: Control, RA-only (50 mg/kg, p.o., 2 days), IFA-only (a single 500 mg/kg, i.p. dose), and IFA + RA. Serum biochemical markers (urea, creatinine), renal oxidative stress parameters (MDA, GSH, SOD, CAT, GPx), gene expression levels (NF-κB, TNF-α, IL-1β, IL-17 A, ACT1, TRAF6, Caspase-3, Bax, Bcl-2, PTGS2, GPX4, TfR1), and histopathological/immunohistochemical analyses (KIM-1, Nephrin, Caspase-3) were performed 24 h post-administration. IFA induced severe renal dysfunction, marked oxidative stress, and extensive histopathological damage. Mechanistically, IFA initiated a pathogenic cascade activating intrinsic apoptosis and ferroptosis, driven by a self-sustaining IL-17 A-TRAF6-NF-κB inflammatory loop. RA co-treatment (50 mg/kg) significantly reversed all functional, biochemical, and histological damage by strategically breaking this crosstalk, restoring redox homeostasis, and simultaneously restraining both cell death programs. In conclusion, RA protects against IFA nephrotoxicity by targeting the critical inflammation-ferroptosis coupling, positioning it as a highly promising adjuvant candidate for clinical use to mitigate IFA-induced renal injury.

## Introduction

Ifosfamide (IFA) remains a critical chemotherapeutic agent within the oxazaphosphorine class [1], widely employed for its potent alkylating activity against a spectrum of solid tumors, including soft-tissue sarcomas, osteosarcoma, and refractory testicular cancers [2]. To exert its cytotoxic effect, IFA must first undergo bioactivation, as it is administered as an inactive prodrug. This metabolic conversion is primarily mediated by hepatic cytochrome P450 (CYP450) isoenzymes, which generate the principal therapeutic metabolite, isophosphoramide mustard (IPM), and the toxic byproduct, chloroacetaldehyde (CAA) [3, 4]. While isophosphoramide mustard is responsible for the antineoplastic activity, CAA is primarily identified as the main mediator of nephrotoxicity due to its accumulation in renal tubular cells, leading to glutathione depletion and oxidative damage [5, 6]. This active compound (IPM) subsequently functions by alkylating DNA, forming inter- and intra-strand cross-links that physically impede DNA replication and transcription, ultimately triggering apoptotic cell death in rapidly proliferating malignant cells [7–9]. The impediment of replication by IPM-induced DNA crosslinks (both intra- and interstrand) leads to caspase-mediated apoptosis, supported by elevated Caspases-3/9 and pro-apoptotic Bcl-2-associated X protein (Bax), and reduced anti-apoptotic B-cell lymphoma 2 (Bcl-2) levels [10–13].

Despite its therapeutic efficacy, the clinical utility of IFA is severely constrained by a high incidence of dose-limiting toxicities, most notably a severe and often irreversible nephrotoxicity. This renal damage is mechanistically distinct from the drug’s antineoplastic effect and is primarily attributed to a separate metabolic byproduct, chloroacetaldehyde (CAA). CAA is known to accumulate preferentially within the renal cortex, particularly in proximal tubule epithelial cells. The mechanism of CAA-induced cytotoxicity is multifactorial but is strongly linked to the direct inhibition of mitochondrial respiratory chain enzymes, particularly Complex I [5, 6]. Clinically, this damage to the tubular epithelium manifests as proximal tubular dysfunction, characterized by proteinuria, glucosuria, and, in severe pediatric cases, Fanconi syndrome [14].

A critical challenge in clinical oncology is the development of adjunctive strategies that can mitigate such organ-specific toxicities without compromising the antineoplastic efficacy of the primary drug [15, 16]. This has fueled intensive research into potential renoprotective agents, with natural phytochemicals emerging as highly promising candidates [17]. Many bioactive compounds derived from edible and medicinal plants, including a wide range of polyphenols, flavonoids, and terpenoids, have been shown to possess potent antioxidant, anti-inflammatory, and anti-apoptotic properties [18–21]. These mechanisms are ideally suited to counteract the specific pathways of chemotherapy-induced damage, such as the oxidative stress, inflammation, and mitochondrial dysfunction initiated by IFA, thereby offering a potential avenue to widen its therapeutic index [22].

Rosmarinic acid (RA) is a phenolic compound found in nature, consisting of an ester linkage between caffeic acid and 3,4-dihydroxyphenyl lactic acid. With a molecular formula of C_18_H_16_O_8_, it is chemically identified as (*R*)-α-[[3-(3,4-dihydroxyphenyl)-1-oxo-2 *E*-propenyl]oxy]-3,4-dihydroxybenzenepropanoic acid [23, 24]. The predominant natural sources of RA include species from the Boraginaceae family, particularly those classified under the Nepetoideae subfamily, as well as members of the Lamiaceae family such as rosemary (*Rosmarinus officinalis*), sage (*Salvia* species), basil (*Ocimum basilicum*), and mint (*Mentha* species), where it serves as a major bioactive phenolic constituent contributing to their antioxidant and therapeutic properties [25–27]. RA has garnered significant scientific interest due to this wide spectrum of robust biological activities. It is well-documented for its potent antioxidant [28, 29], anti-inflammatory [30], antimicrobial [31], anti-apoptotic [32, 33], antidiabetic [34], and nephroprotective [35, 36] properties. Mechanistically, RA is known to exert these protective effects through several interconnected pathways. These include the direct scavenging of reactive oxygen species (ROS), chelation of pro-oxidant metals, and the preservation of mitochondrial integrity. Concurrently, RA modulates key inflammatory signaling pathways, notably through the suppression of Nuclear factor kappa B (NF-κB) and the inhibition of the NOD-like receptor family pyrin domain containing 3 (NLRP3) inflammasome. These established mechanisms directly align with the known drivers of chemotherapy-induced toxicity [37–39].

While previous studies have demonstrated the protective efficacy of RA against various other nephrotoxic agents [35, 40, 41], its specific protective effect against IFA-induced nephrotoxicity remains unelucidated. To our knowledge, no study has yet investigated the molecular mechanisms by which RA might counteract the specific pathogenic pathways initiated by IFA’s toxic metabolite, CAA, particularly concerning the critical interplay between inflammation, apoptosis, and the emerging pathway of ferroptosis.

Although the nephroprotective potential of RA has been reported against various agents such as cisplatin and methotrexate, to date, no study has investigated its specific protective efficacy against IFA-induced nephrotoxicity. Furthermore, this study is the first to elucidate a novel mechanistic link by which RA targets the Interleukin-17 A (IL-17 A) / NF-κB activator 1 (ACT1) / TNF receptor-associated factor 6 (TRAF6) axis to disrupt the inflammation-ferroptosis coupling, distinguishing it from previous oxidative stress-focused investigations. Therefore, the primary objective of this study was to investigate the potential renoprotective effects of RA against IFA-induced kidney damage in a rat model. We aimed to elucidate the specific molecular mechanisms of this protection, focusing on the pathogenic crosstalk between apoptosis, Glutathione peroxidase 4 (GPX4) and Transferrin receptor 1 (TfR1)-mediated ferroptosis, and the subsequent NF-κB-driven inflammatory cascade. A novel focus of this study was to determine if RA’s efficacy stems from its ability to disrupt the IL-17 A/TRAF6 signaling pathway, which we identified as a key amplification loop linking these initial cell death pathways to sustained inflammation. By evaluating this complex interplay, we assessed whether RA could mitigate the downstream physical damage, characterized by glomerular and tubular injury, thereby preserving overall renal architecture and function.

##  Materials and methods

### Reagents and chemicals

IFA (CAS: 3778-73-2) and RA (CAS: 20283-92-5) were purchased from Sigma-Aldrich, St. Louis, MO, USA. All other analytical-grade chemicals and reagents were procured from commercial sources.

### Animal husbandry and ethical approval

This investigation utilized twenty-eight [28] adult male *Wistar albino* rats (aged 7–9 weeks), weighing between 230 and 250 g (mean weight: 240 ± 10 g). The animals were supplied by the Necmettin Erbakan University Experimental Medicine Application and Research Center (KONÜDAM, Konya, Türkiye). Throughout the study, rats were maintained in standard polycarbonate cages under controlled environmental conditions with unrestricted access to standard laboratory chow and tap water ad libitum. The housing facility was maintained at a stable temperature of 22 ± 2 °C, 50–60% relative humidity, and a 12-hour light/dark photoperiod (lights on at 07:00 h). Prior to the experimental procedures, all animals underwent a 7-day acclimatization period. The entire experimental protocol was reviewed and approved by the Necmettin Erbakan University Animal Experiments Local Ethics Committee (KONÜDAM-HADYEK) (Approval Date/Number: 2025-063). All procedures were conducted in strict accordance with the Guide for the Care and Use of Laboratory Animals and the ARRIVE guidelines.

### Experimental design and treatment protocol

IFA and RA solutions were freshly prepared in sterile saline immediately before administration. To ensure precise dosing for each animal, the injection volume was calculated based on the individual body weight.

Following the acclimatization period, the animals were randomly allocated into four experimental groups (*n* = 7 per group) using a computer-generated randomization sequence:

Group 1 (Control): Animals received 0.9% sterile saline solution via both oral gavage (p.o., 1 mL) and intraperitoneal injection (i.p., 1 mL) once daily for 2 consecutive days.

Group 2 (IFA): Animals received 0.9% sterile saline solution via oral gavage (p.o., 1 mL) once daily for 2 days. On the second day, immediately following the oral saline administration, a single dose of IFA (500 mg/kg body weight, dissolved in sterile saline) was administered via intraperitoneal injection (i.p.).

Group 3 (RA): Animals received RA (50 mg/kg body weight, dissolved in sterile saline) via oral gavage (p.o.) once daily for 2 consecutive days. On the second day, immediately following the RA administration, animals received 0.9% sterile saline solution via intraperitoneal injection (i.p., 1 mL).

Group 4 (IFA + RA): Animals received RA (50 mg/kg body weight, p.o.) once daily for 2 consecutive days. On the second day, immediately following the RA administration, a single dose of IFA (500 mg/kg body weight, i.p.) was administered.

The dose of IFA (500 mg/kg, i.p.) was selected based on previous studies demonstrating significant nephrotoxicity in rodent models [42]. The dose of RA (50 mg/kg, p.o.) was chosen according to prior investigations showing optimal protective effects against chemotherapy-induced organ toxicity without adverse effects [43]. All administrations were performed between 09:00 and 11:00 h to minimize circadian variations.

### Sample collection and processing

Twenty-four hours after the final administration (on Day 3), all animals were weighed and anesthetized using sevoflurane inhalation (5% for induction, 2–3% for maintenance). Following deep anesthesia confirmation (absence of pedal withdrawal reflex), animals were sacrificed via decapitation. Blood samples (approximately 3–5 mL) were immediately collected into non-heparinized vacuum tubes and allowed to clot at room temperature for 30 min. The samples were then centrifuged at 3000 rpm (approximately 1000 g) for 10 min at + 4 °C to separate the serum. The obtained serum was carefully transferred into sterile microcentfuge tubes and stored at -80 °C until biochemical analysis.

Both kidneys were rapidly excised, rinsed with ice-cold phosphate-buffered saline (PBS, pH 7.4) to remove residual blood, and blotted dry on filter paper. The kidneys were weighed, and the kidney-to-body weight ratio was calculated. The left kidney from each animal was immediately snap-frozen in liquid nitrogen and stored at -80 °C for subsequent biochemical and molecular analyses. The right kidney from each animal was immediately fixed in 10% neutral-buffered formalin solution for 24 h. Following fixation, the tissues were processed through standard dehydration steps and embedded in paraffin. These formalin-fixed, paraffin-embedded blocks were subsequently sectioned for both histopathological and immunohistochemical analyses.

### Biochemical analyses

#### Assessment of renal function

Renal functional status was evaluated by quantifying serum urea and creatinine concentrations. These measurements were conducted utilizing commercially available diagnostic kits on a Mindray Perfect Plus 400 automated biochemical analyzer (Mindray Medical International Limited, Shenzhen, China), strictly adhering to the protocols provided by the manufacturer [44, 45].

#### Evaluation of Pro-Oxidant-Antioxidant balance

Kidney tissue specimens were mechanically disrupted using a TissueLyser II homogenizer (Qiagen, Hilden, Germany) and subsequently homogenized in ice-cold 1.15% potassium chloride buffer solution (pH 7.4). Following homogenization, the samples underwent centrifugation at 12,298 x g for 20 min at 4 °C, and the clarified supernatants were collected and utilized for all subsequent oxidative stress-related biochemical determinations. The extent of lipid peroxidation was assessed by spectrophotometric quantification of malondialdehyde (MDA) concentration at 532 nm wavelength, utilizing the thiobarbituric acid reactive substances (TBARS) assay as originally described by Placer and colleagues [46]. The antioxidant defense capacity of renal tissues was comprehensively characterized by determining the enzymatic activities of superoxide dismutase (SOD), catalase (CAT), and glutathione peroxidase (GPx), in addition to measuring reduced glutathione (GSH) content. Specifically, CAT enzymatic activity was measured following the methodology established by Aebi [47], SOD activity was assessed according to the protocol described by Sun and coworkers [48], GPx activity was determined using the procedure outlined by Lawrence and Burk [49], and GSH concentrations were quantified based on the method reported by Sedlak and Lindsay [50]. To ensure accurate comparison across samples, all enzymatic activities were normalized against total protein concentration, which was determined spectrophotometrically using the classical Lowry method [51].

### Total RNA extraction and cDNA Preparation from renal tissue specimens

To examine the transcriptional profiles of target genes across all experimental cohorts, total ribonucleic acid (RNA) was extracted from kidney tissue specimens immediately following collection. RNA isolation was accomplished using QIAzol Lysis Reagent (Cat. No. 79306, Qiagen, Hilden, Germany), with strict adherence to the extraction protocol recommended by the supplier. The quality and purity of the isolated RNA were assessed via spectrophotometric measurement, specifically by evaluating the absorbance ratio at 260/280 nm using a NanoDrop spectrophotometer (Thermo Fisher Scientific, Waltham, MA, USA). Additionally, the RNA concentration for each specimen was quantified and recorded before proceeding to reverse transcription. First-strand complementary DNA (cDNA) synthesis was subsequently performed using the QuantiTect Reverse Transcription Kit (Cat. No. 330411, Qiagen, Germany), following both the manufacturer’s recommended procedure and established methodologies documented in prior literature [52]. The nucleotide sequences of primers designed for amplification of all investigated genes are provided in Table 1. Real-time quantitative polymerase chain reaction (qPCR) experiments were conducted with a minimum of three technical replicates for each target gene to guarantee data reliability and reproducibility. The housekeeping gene β-actin was employed as an endogenous reference control to normalize variations in gene expression levels among samples. Threshold cycle (Ct) values derived from the amplification reactions were analyzed using the comparative 2^−ΔΔCt^ method as established by Livak and Schmittgen [53] thereby facilitating the calculation of relative mRNA expression levels for each gene of interest across the experimental groups.

**Table 1:** Primer sequences

Gene	Sequences (5’-3’)	Accession No
**NF-κB**	F: AGTCCCGCCCCTTCTAAAAC R: CAATGGCCTCTGTGTAGCCC	NM_001276711.1
**TNF-α**	F: CTCGAGTGACAAGCCCGTAG R: ATCTGCTGGTACCACCAGTT	NM_012675.3
**IL-1β**	F: ATGGCAACTGTCCCTGAACT R: AGTGACACTGCCTTCCTGAA	NM_031512.2
**IL17A**	F: TGCCTGATGCTGTTGCTGCTAC R: TTGGACACACTGAACTTTGAGGGATG	NM_001106897.1
**ACT1**	F: GAAGCATTCCCGTGGAGGTTGAC R: TGGGTGCCGTGTTCCTTGTATTTG	XM_039098886.1
**TRAF6**	F: GTGCGTCCAGCCAGTCTTCAAG R: AGCCACTCACACTGTCATCTTTCATG	NM_001107754.2
**Caspase-3**	F: ACTGGAATGTCAGCTCGCAA R: GCAGTAGTCGCCTCTGAAGA	NM_012922.2
**Bax**	F: TTTCATCCAGGATCGAGCAG R: AATCATCCTCTGCAGCTCCA	NM_017059.2
**Bcl-2**	F: GACTTTGCAGAGATGTCCAG R: TCAGGTACTCAGTCATCCAC	NM_016993.2
**PTGS2**	F: CTCAGCCATGCAGCAAATCC R: GGGTGGGCTTCAGCAGTAAT	NM_017232
**GPX4**	F: TCTGAGCCGCTTATTGAAGCC R: CACACGCAACCCCTGTACTT	NM_017165
**TfR1**	F: CCGGCCTATATGCTTGGGTA R: CAAGGGAGCACTCTGAAGCA	NM_022712.1
**β-Actin**	F: CAGCCTTCCTTCTTGGGTATG R: AGCTCAGTAACAGTCCGCCT	NM_031144.3

### Histopathological examination

#### Hematoxylin and Eosin staining

Collected kidney tissue specimens were fixed in 10% neutral-buffered formalin solution for 24 h. Following fixation, the tissues were processed through a standard paraffin tissue processing protocol, involving sequential dehydration through graded alcohols, clearing in xylene, and infiltration with molten paraffin wax, before being embedded in paraffin blocks. Serial sections of 5 μm thickness were obtained from the paraffin blocks using a rotary microtome (Leica RM2125 RTS, Leica Biosystems, Wetzlar, Germany) and mounted onto glass slides. For histological evaluation, sections were stained with hematoxylin and eosin (H&E) according to standard protocols. The stained sections were examined under a CX43 light microscope (Olympus Corporation, Tokyo, Japan), and digital images were captured using an EP50 camera (Olympus Corporation). Micrographs were obtained at 200× and 400× magnifications. To minimize observer bias, renal tissue sections were evaluated using a blinded methodology during microscopic examination. Tissue damage was assessed based on the presence and severity of glomerular vacuolization, tubular dilatation, inflammatory cell infiltration, and vascular congestion. Each parameter was scored according to the following semi-quantitative scoring system: 0 = no damage, 1 = < 25%, 2 = 26–50%, 3 = 51–74%, and 4 = ≥ 75% damage. For each sample, six randomly selected fields were analyzed, and the mean scores were calculated [54].

#### Immunohistochemical analysis

Paraffin-embedded kidney tissue blocks were sectioned at 3 μm thickness and prepared for immunohistochemical analysis. Heat-induced antigen retrieval was performed by immersing the sections in citrate buffer (pH 6.0) and heating in a microwave oven. Subsequently, to quench endogenous peroxidase activity, sections were incubated in 0.3% hydrogen peroxide solution in the dark for 10 min at room temperature. Sections were then incubated with primary antibodies in a humidified chamber. The following primary antibodies were applied: T-cell immunoglobulin and mucin domain 1 (TIM-1) / Kidney injury molecule-1 (KIM-1) (Cat. No. PA1632; Boster Biological Technology, Pleasanton, CA, USA), Nephrin (Cat. No. PA5-20330; Thermo Fisher Scientific, Waltham, MA, USA), and Caspase-3 (Cat. No. SC-56053; Santa Cruz Biotechnology, Dallas, TX, USA), each diluted at a ratio of 1:100 in antibody diluent. The slides were incubated overnight at + 4 °C. The following day, sections were incubated with biotinylated secondary antibody, followed by streptavidin-horseradish peroxidase (HRP) complex, to complete the binding process. Immunoreactivity was visualized using 3,3’-diaminobenzidine tetrahydrochloride (DAB Substrate System, Ready-to-Use; Thermo Fisher Scientific) as the chromogen. For contrast staining, sections were counterstained with hematoxylin for 5 min, dehydrated through a graded series of alcohols, cleared in xylene, and coverslipped with mounting medium. Stained sections were examined under an Olympus CX43 binocular light microscope (Olympus Corporation, Tokyo, Japan) and photographed at 400x magnification using an integrated digital camera. To ensure objective analysis, the staining intensity was quantitatively assessed using ImageJ software (version 1.46a; National Institutes of Health, Bethesda, MD, USA) by calculating the percentage of positive staining area in five randomly selected tissue sections from each experimental group.Immunohistochemical staining intensity was also evaluated using a semi-quantitative scoring system ranging from 0 to 3 (0 = no staining, 1 = weak, 2 = moderate, 3 = strong staining).

### Statistical analysis

All experimental data were analyzed using appropriate statistical methods. Results are presented as mean ± standard deviation (SD). Statistical comparisons among experimental groups were conducted using one-way analysis of variance (ANOVA), followed by Tukey’s post hoc test to identify specific inter-group differences when ANOVA indicated statistical significance. A probability value of *p* < 0.05 was considered to indicate statistical significance. Data analysis and graphical representation were performed using GraphPad Prism software (version 5.01; GraphPad Software Inc., San Diego, CA, USA).

## Results

### RA ameliorates IFA-Induced renal dysfunction

**Fig. 1:**
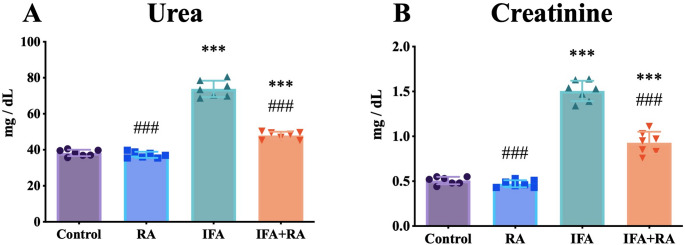
Effects of IFA and RA administrations on kidney function markers: (**A**) Serum urea and (**B**) Serum creatinine. Values are given as mean ± SD. Statistical significance (Control vs. others: *p ˂ 0.05, **p ˂ 0.01, ***p < 0.001, IFA vs. others: #p ˂ 0.05, ##p ˂ 0.01, ###p ˂ 0.001, ns: not significant) was analyzed using One-Way ANOVA

Administration of IFA resulted in a 93.0% increase in serum urea (73.85 ± 4.54 mg/dL) compared to the control group (38.27 ± 1.78 mg/dL) (*p* < 0.001). Co-treatment with RA (IFA + RA group) significantly reduced urea levels to 47.92 ± 2.09 mg/dL, a 35.1% reduction versus the IFA group (*p* < 0.001) and corresponding to a 72.9% recovery toward control. Similarly, serum creatinine increased by 196.9% in the IFA group (1.51 ± 0.11 mg/dL) versus control (0.51 ± 0.04 mg/dL) (*p* < 0.001). The IFA + RA group showed significantly lowered creatinine (0.93 ± 0.12 mg/dL), a 38.3% reduction versus IFA (*p* < 0.001) and a 57.8% recovery. The RA-only group (37.21 ± 1.70 mg/dL urea; 0.47 ± 0.04 mg/dL creatinine) did not differ significantly from the control group (*p* > 0.05) (Fig. 1).

### RA restores antioxidant defenses and mitigates lipid peroxidation

**Fig. 2:**
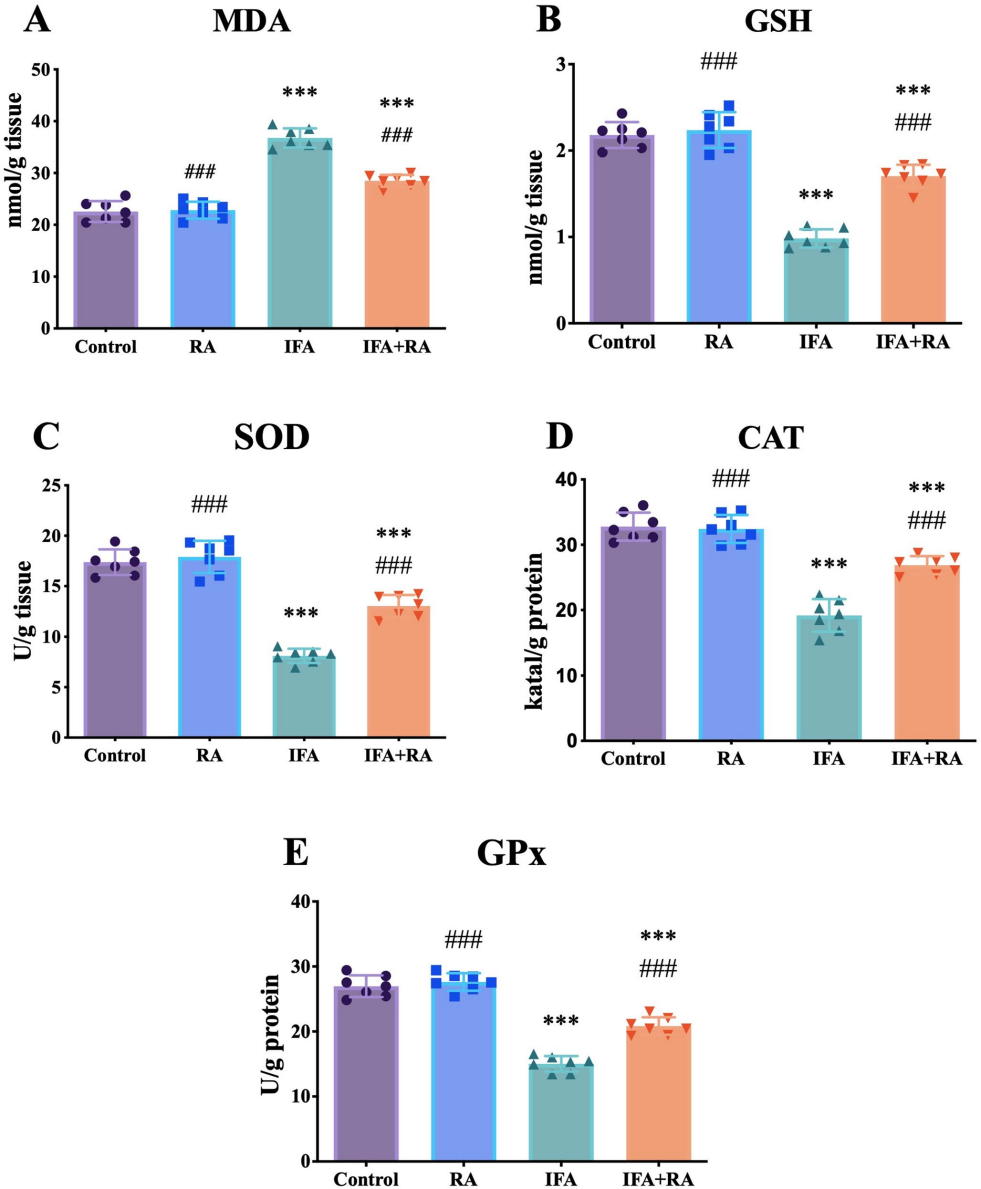
Effects of IFA and RA administrations on oxidative stress markers: (**A**) MDA, (**B**) GSH, (**C**) SOD, (**D**) CAT, and (**E**) GPx. Values are given as mean ± SD. Statistical significance (Control vs. others: *p ˂ 0.05, **p ˂ 0.01, ***p < 0.001, IFA vs. others: #p ˂ 0.05, ##p ˂ 0.01, ###p ˂ 0.001, ns: not significant) was analyzed using One-Way ANOVA

IFA administration increased MDA levels by 62.9% (36.77 ± 1.85 nmol/mg protein) compared to control (22.57 ± 2.01 nmol/mg protein) (*p* < 0.001). This was significantly reduced by 22.5% in the IFA + RA group (28.49 ± 1.15 nmol/mg protein) (*p* < 0.001 vs. IFA), achieving a 58.3% recovery. IFA also caused a 54.9% depletion of GSH (0.98 ± 0.11 µmol/mg protein), a 53.3% reduction in SOD (8.11 ± 0.70 U/mg protein), a 41.4% reduction in CAT (19.21 ± 2.48 U/mg protein), and a 44.3% reduction in GPx (15.02 ± 1.20 U/mg protein) (all *p* < 0.001 vs. control). RA co-treatment significantly reversed these depletions, increasing GSH by 73.4% (1.70 ± 0.13 µmol/mg protein), SOD by 60.7% (13.03 ± 1.09 U/mg protein), CAT by 39.9% (26.88 ± 1.39 U/mg protein), and GPx by 38.7% (20.83 ± 1.35 U/mg protein) (all *p* < 0.001 vs. IFA). The RA-only group was not significantly different from control for any redox parameter (*p* > 0.05) (Fig. 2).

### RA modulates gene expression of pathogenic pathways

#### Canonical inflammatory axis (NF‑κB/TNF‑α/IL‑1β)

**Fig. 3:**
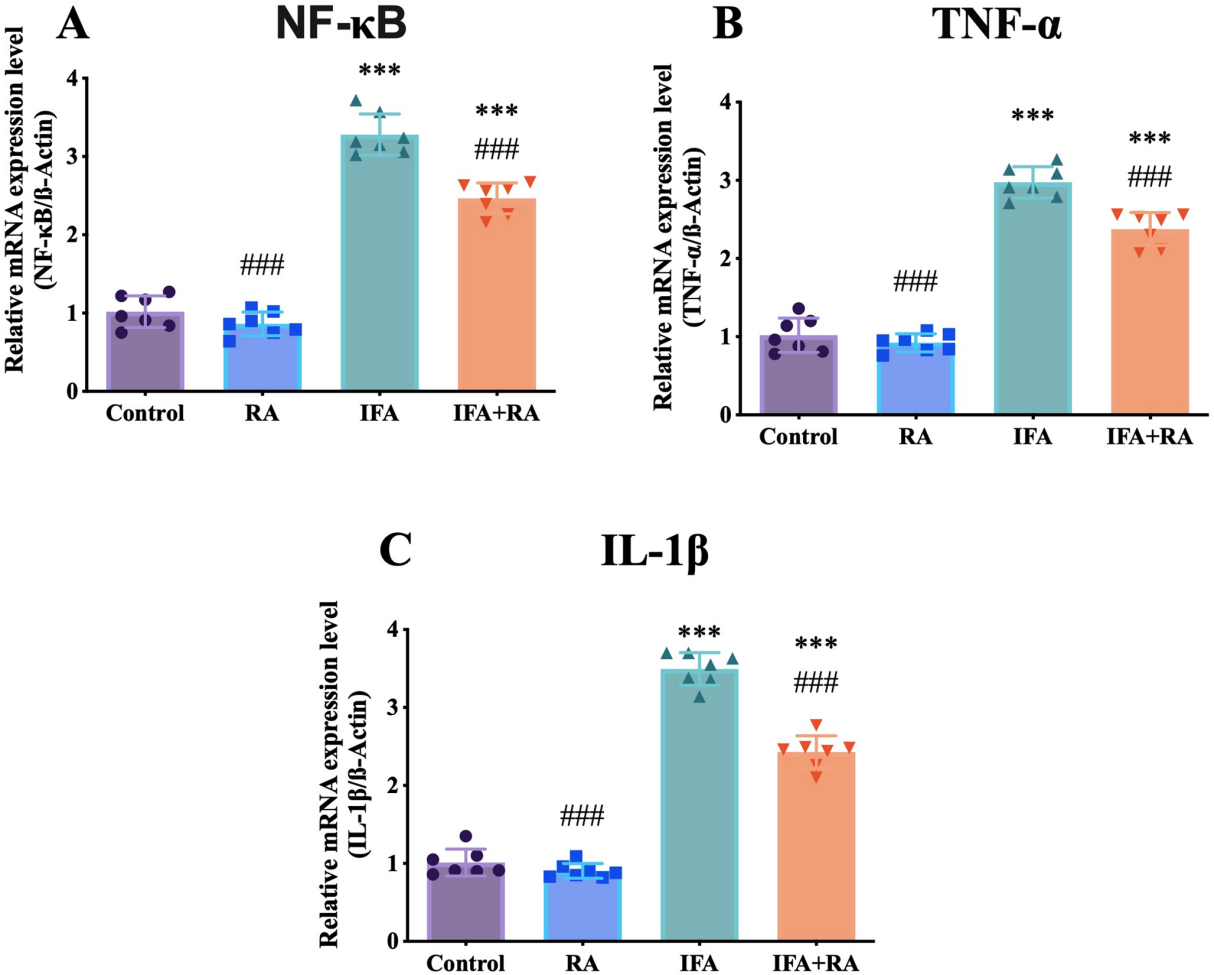
Effects of IFA and RA administrations on inflammatory markers: (**A**) NF-κB, (**B**) TNF‑α, and (**C**) IL‑1β. Values are given as mean SD. Statistical significance (Control vs. others: *p ˂ 0.05, **p ˂ 0.01, ***p < 0.001, IFA vs. others: #p ˂ 0.05, ##p ˂ 0.01, ###p ˂ 0.001, ns: not significant) was analyzed using One-Way ANOVA

For inflammatory markers, IFA induced a 222.3% (3.22-fold) increase in NF-κB (3.28 ± 0.26), which was reduced by 24.8% in the IFA + RA group (2.47 ± 0.20). Tumor Necrosis Factor-alpha (TNF-α) was increased by 192.0% (2.97 ± 0.20), and reduced by 20.1% in the IFA + RA group (2.38 ± 0.21). Interleukin-1 beta (IL-1β) was increased by 245.5% (3.49 ± 0.21), and reduced by 30.5% in the IFA + RA group (2.43 ± 0.21) (Fig. 3).

#### IL‑17 A/ACT1/TRAF6 amplification loop

**Fig. 4:**
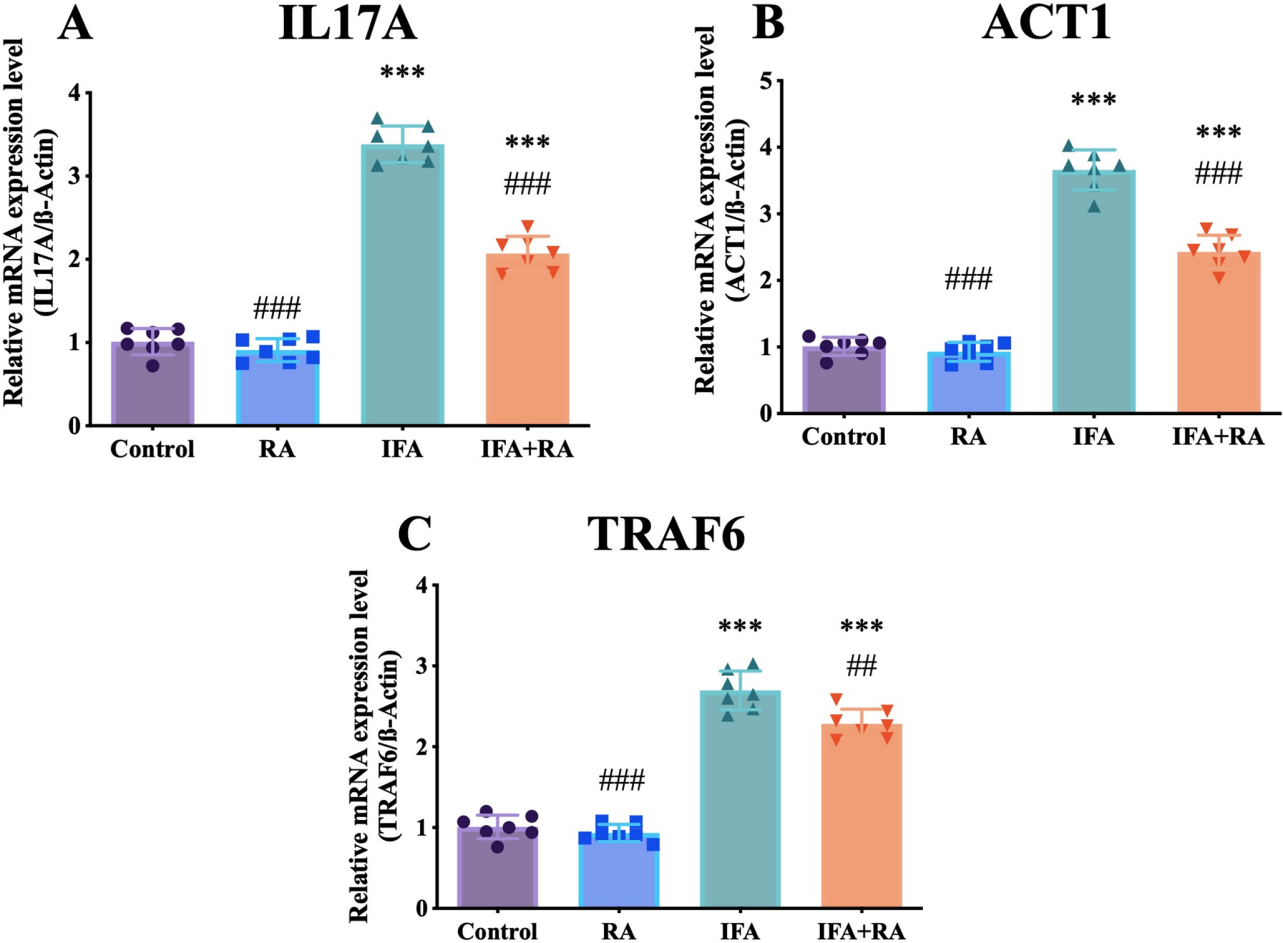
Effects of IFA and RA administrations on IL-17 A signaling pathway markers: (**A**) IL-17 A, (**B**) ACT1, and (**C**) TRAF6. Values are given as mean SD. Statistical significance (Control vs. others: *p ˂ 0.05, **p ˂ 0.01, ***p < 0.001, IFA vs. others: #p ˂ 0.05, ##p ˂ 0.01, ###p ˂ 0.001, ns: not significant) was analyzed using One-Way ANOVA

IL-17 A signaling pathway was similarly affected: IL-17 A (+ 234.8%, 3.38 ± 0.22), ACT1 (+ 262.7%, 3.66 ± 0.30), and TRAF6 (+ 167.4%, 2.70 ± 0.24) were all upregulated by IFA and significantly, though partially, reduced by RA co-treatment (by 38.8%, 33.7%, and 15.4%, respectively) (Fig. 4).

#### Intrinsic apoptosis(Caspase–3/Bax/Bcl–2)

**Fig. 5:**
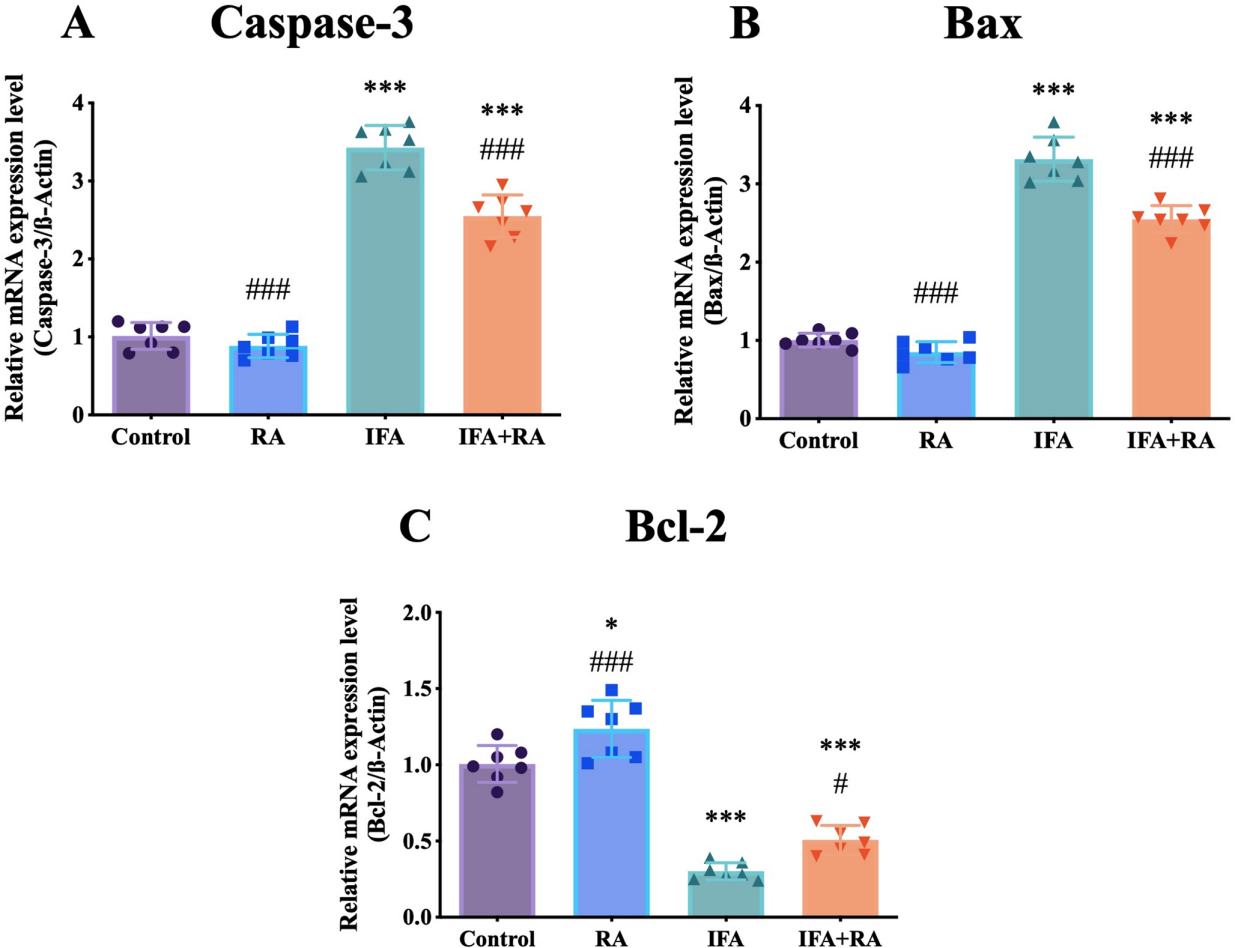
Effects of IFA and RA administrations on apoptotic markers: (**A**) Caspase-3, (**B**) Bax, and (**C**) Bcl-2. Values are given as mean SD. Statistical significance (Control vs. others: *p ˂ 0.05, **p ˂ 0.01, ***p < 0.001, IFA vs. others: #p ˂ 0.05, ##p ˂ 0.01, ###p ˂ 0.001, ns: not significant) was analyzed using One-Way ANOVA

For apoptotic markers, IFA upregulated Caspase-3 by 238.4% (3.43 ± 0.28) and Bax by 230.2% (3.32 ± 0.28), while suppressing Bcl-2 by 70.0% (0.30 ± 0.06). RA co-treatment reduced Caspase-3 and Bax expression by 25.6% and 23.2%, respectively, and significantly increased Bcl-2 expression by 68.2% (0.51 ± 0.09) (*p* < 0.05 vs. IFA) (Fig. 5).

#### Dysregulation of the GPX4/TfR1 axis and PTGS2 expression

**Fig. 6:**
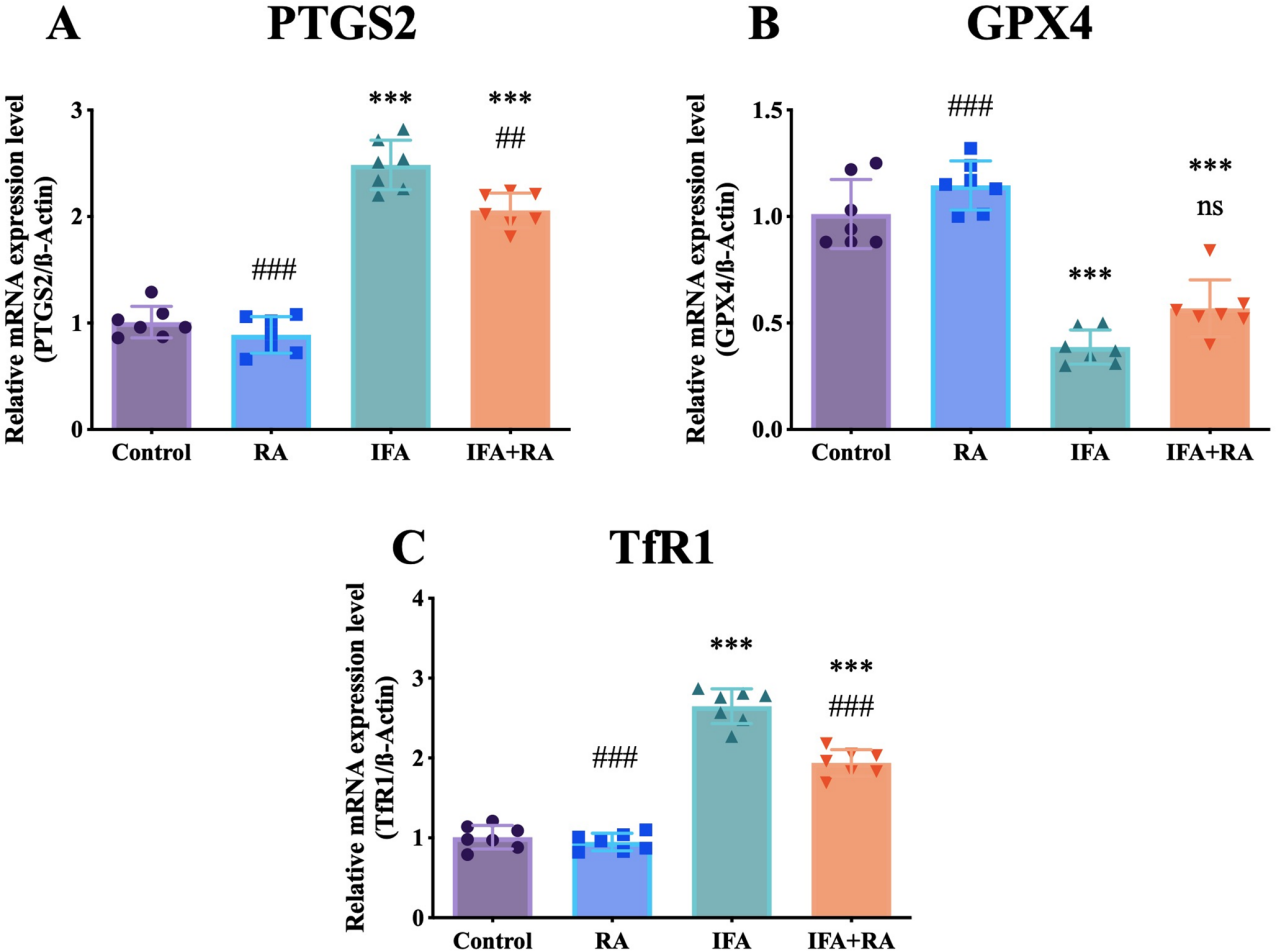
Effects of IFA and RA administrations on ferroptosis-related markers: (**A**) PTSG2, (**B**) GPX4 and (**C**) TfR1. Values are given as mean SD. Statistical significance (Control vs. others: *p ˂ 0.05, **p ˂ 0.01, ***p < 0.001, IFA vs. others: #p ˂ 0.05, ##p ˂ 0.01, ###p ˂ 0.001, ns: not significant) was analyzed using One-Way ANOVA

For ferroptosis-related markers, IFA induced a 146.3% upregulation of Prostaglandin-Endoperoxide Synthase 2 (PTGS2) (2.48 ± 0.23) (*p* < 0.001 vs. control), which was also significantly, though partially, reduced by RA co-treatment (2.06 ± 0.16) (*p* < 0.05 vs. IFA). IFA also suppressed GPX4 by 61.7% (0.39 ± 0.08) and upregulated TfR1 by 162.6% (2.65 ± 0.22) (*p* < 0.001 vs. control). RA co-treatment significantly reversed these latter two markers, increasing GPX4 by 46.9% (0.57 ± 0.13) and decreasing TfR1 by 26.8% (1.94 ± 0.17) (both *p* < 0.001 vs. IFA) (Fig. 6).

### Histological and immunohistochemical analysis

####  Histopathological Evaluation

**Fig. 7:**
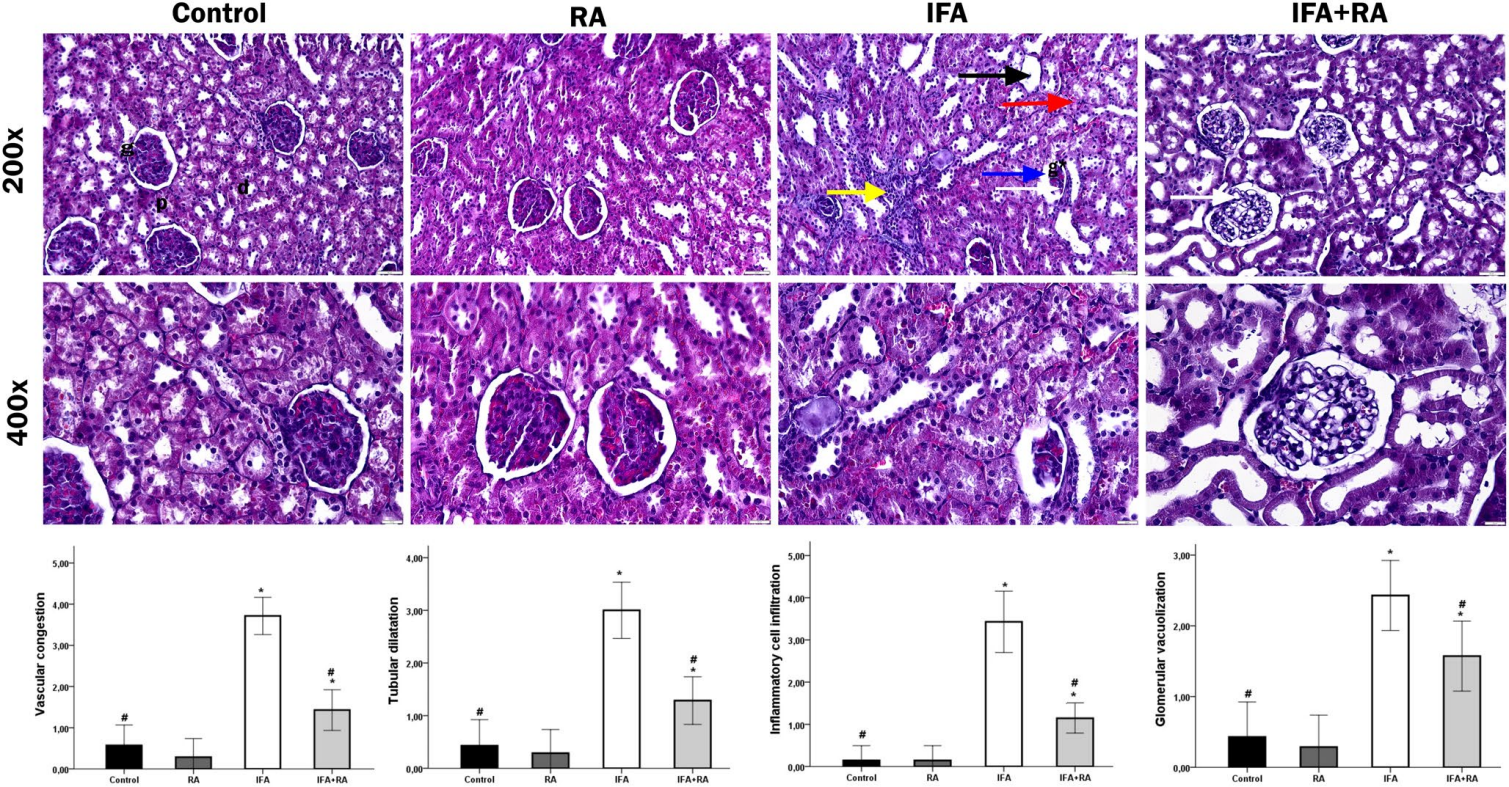
H&E-stained histological sections of renal tissues from experimental groups (×200, ×400). The Control and RA groups exhibited regular morphology characterized by normal glomeruli (g) surrounded by Bowman's capsule, proximal convoluted tubules (p) with narrow lumens, and distal convoluted tubules (d) with wide lumens. In the IFA group, markedly shrunken and atrophic glomeruli (g*) were noted, accompanied by consequent dilatation of Bowman’s space (blue arrow). Additional findings included tubular dilation in proximal and distal tubules (black arrow), focal loss of epithelial cells, vacuolization in podocytes and certain tubular epithelial cells (white arrow), increased interstitial inflammatory cell infiltration (yellow arrow), and extravasation of erythrocytes into the renal lumen (red arrow). The IFA+RA group demonstrated partial recovery and a histological structure resembling the control group, with the exception of mild hyperemia and a reduced presence of vacuolated cells (white arrow). Note: Data represent mean ± SD. *p<0.05 compared to control groups, #p<0.05 compared to the IFA group.

The H&E staining results for the experimental groups are presented in Fig. 7. In the kidney tissue sections from the Control and RA groups, the glomeruli in the cortex, as well as the tubules and medullary structures, exhibited a regular morphology. Notably, no degenerative changes were observed in the proximal and distal tubule cells. In contrast, in the IFA-administered group, tubular structures were locally dilated, epithelial cells of the tubules showed desquamation, and vacuolization was detected in both the tubular epithelial cells and podocytes. Furthermore, interstitial inflammatory cell infiltration, interstitial hemorrhage, and vascular congestion were observed. In the IFA + RA group, the histological abnormalities in the glomerular and tubular components were markedly corrected by RA administration. However, it was found that a few tubules still exhibited dilatation, and some cells with vacuolization were still observed in some glomeruli and tubules. According to the histopathological scoring results, while the mean damage score was low in the control group, this score was statistically significantly increased by IFA (*p* < 0.05). In contrast, the mean score in the IFA + RA group showed a significant decrease compared to the IFA group (*p* < 0.05).

#### Immunohistochemical analysis of renal injury and apoptosis

**Fig. 8:**
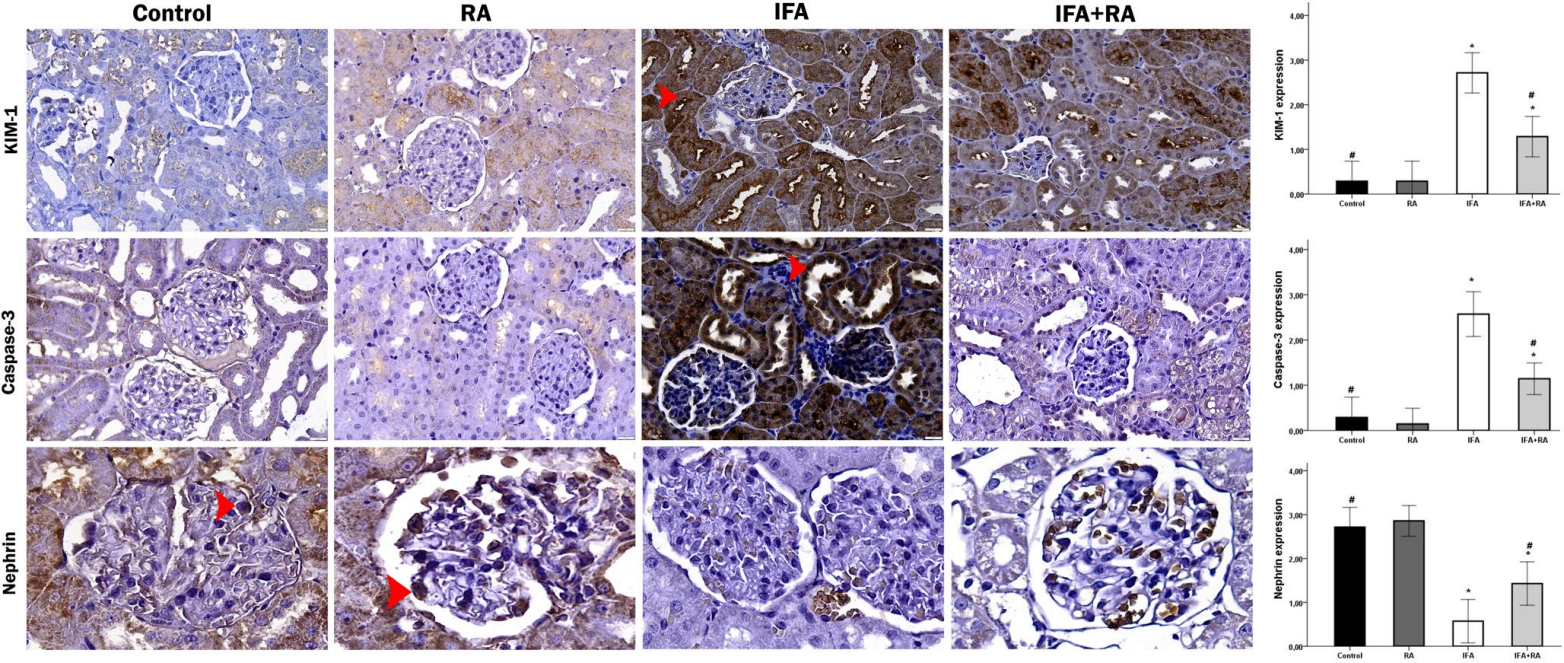
Changes in KIM-1/TIM-1, Caspase-3, and Nephrin protein expression levels in renal tissues across different experimental groups. In the Control and RA groups, KIM-1/TIM-1 and Caspase-3 staining was minimal, showing only faint immunohistochemical reactivity, whereas Nephrin expression exhibited a strong positive reaction (arrowhead). Conversely, the IFA group displayed markedly increased KIM-1/TIM-1 and Caspase-3 expression with intense positive staining (arrowhead), while Nephrin expression was significantly diminished. In the IFA+RA group, a reduction in KIM-1/TIM-1 and Caspase-3 expression and a partial increase in Nephrin staining were observed compared to the IFA group, with generally moderate positive reactions detected. IHC, DAB chromogen, hematoxylin counterstain; ×400 magnification. Note: Data represent mean ± SD. *p<0.05 compared to control groups, #p<0.05 compared to the IFA group.

The immunohistochemical expression of KIM-1, Caspase-3, and Nephrin proteins was evaluated in kidney tissue sections, and the results are presented in Fig. 8. KIM-1 staining was predominantly observed localized to the proximal tubular epithelial cells. While KIM-1 expression was minimal in the renal tissues of the Control and RA groups, a significant increase was detected in the IFA-administered group. No specific staining was observed in glomerular or interstitial cells. According to the semi-quantitative analysis results, the percentage of KIM-1 positive cells was statistically significantly higher in the IFA group compared to the control group (*p* < 0.05). In the IFA + RA group, the percentage of KIM-1 positive cells was significantly decreased compared to the IFA group (*p* < 0.05). When Caspase-3 expression was evaluated by immunohistochemistry, positive staining was found to be very weak in the control and RA groups, with only a mild level of positivity detected in a limited number of cells. On the other hand, a marked increase in Caspase-3 expression was observed in the IFA group. Widespread and intense cytoplasmic DAB staining was seen in the tubule epithelial cells and in the podocytes within the glomeruli. According to the semi-quantitative scoring results, while the Caspase-3 staining score was minimal in the control and RA groups, the Caspase-3 intensity was determined to be statistically significantly increased in the IFA group (*p* < 0.05). In the IFA + RA group, a significant decrease in Caspase-3 expression was recorded compared to the IFA group (*p* < 0.05). Nephrin staining showed an intense distribution localized to the podocytes in the glomeruli. In the Control and RA groups, nephrin expression was observed regularly and distinctly along the glomerular structures. In contrast, a marked decrease in nephrin expression was observed in the IFA group. Irregular and weak staining around the glomerulus was noted; in some areas, nephrin expression was completely lost. According to the scoring evaluation, staining levels were markedly decreased in the IFA group. In the IFA + RA group, nephrin expression was significantly increased compared to the IFA group (*p* < 0.05).

## Discussion

RA, a natural polyphenol with potent antioxidant and anti-inflammatory properties, has garnered significant attention as a potential protective agent against such chemotherapy-induced toxicities [55]. IFA is a widely used and effective antineoplastic agent, yet its clinical efficacy is frequently overshadowed by severe, dose-limiting nephrotoxicity [56]. The present study elucidates the core protective mechanism of RA against this CAA-mediated nephrotoxicity, demonstrating for the first time that RA strategically limits the inflammation–ferroptosis coupling by disrupting the IL-17 A-TRAF6 crosstalk. This protective effect was consistently verified across functional, biochemical, molecular, and histological levels.

Urea and creatinine are the most reliable biochemical indicators of glomerular filtration and tubular function; their acute elevation reflects impairment in the filtration barrier and/or proximal tubular function [57, 58]. The present findings demonstrated that IFA markedly elevated these two parameters, whereas RA co-treatment significantly attenuated these increases, achieving a substantial recovery toward control levels. This pattern is consistent with the established clinical and experimental manifestations of nephrotoxicity mediated by IFA’s primary metabolite, CAA [59]. The fact that RA alone did not alter these indicators supports its lack of inherent nephrotoxicity, suggesting that the observed benefit stems directly from the modulation of IFA-specific pathology. These results are thus in alignment with the broader nephroprotector literature, which has previously reported the capacity of RA to drive functional recovery in chemotherapy-induced renal injury [60–64].

MDA is the end product of lipid peroxidation and is considered an indicator of membrane damage, whereas GSH, along with SOD, CAT, and GPx activities, are the fundamental components of the cellular antioxidant defense [65–68]. The finding that IFA increased MDA while decreasing GSH, SOD, CAT, and GPx is consistent with the mechanism by which CAA increases ROS production through the suppression of the mitochondrial respiratory chain [69]. RA significantly reduced MDA while elevating the antioxidant defense (the GSH pool and SOD/CAT/GPx activities), thereby restoring redox homeostasis at a foundational level. This finding overlaps with studies reporting the direct free-radical scavenging, metal-chelating, and mitochondria-protective effects of RA [70–72], and prepares the biological basis for the attenuation of downstream inflammation and cell death programs. Indeed, it has been previously shown that RA mitigates oxidative damage in chemotherapy-induced organ toxicities [73–75].

NF-κB is the principal driver of proinflammatory transcription in the renal parenchyma, while TNF-α and IL-1β are key effector cytokines that potentiate endothelial–epithelial interactions and leukocyte infiltration [76, 77]. The IFA-induced increases in NF-κB and in TNF-α and IL-1β indicate a robust inflammatory microenvironment precipitated by oxidative stress. RA significantly attenuated this axis, thereby lowering the cytokine burden and the risk of secondary tissue injury. RA’s anti-inflammatory effects on NF-κB/NLRP3 are well documented across multiple models [23, 36, 78–80], and our findings suggest that a similar suppression occurs in IFA-induced nephrotoxicity. Considered alongside the weakening of the IL-17 A/ACT1/TRAF6 axis discussed below, this suppression suggests that the self-perpetuating nature of inflammation is concurrently disrupted at upstream and downstream levels.

The engagement of TRAF6 by IL-17 A signaling via ACT1 amplifies IKK/NF-κB activation and converts the canonical axis into a self-sustaining inflammatory loop [81, 82]. IFA-induced upregulation of IL-17 A, ACT1, and TRAF6 demonstrates that this amplification circuit actively contributes to pathogenesis. RA attenuated this amplifier by inducing significant reductions in all three markers (particularly > 30% in IL-17 A and ACT1), thereby targeting not only the downstream outputs of NF-κB (cytokines) but also the upstream signaling that sustains it. When considered alongside evidence that RA suppresses inflammatory axes (NF-κB/NLRP3) [83], our findings at the IL-17 A node suggest that RA substantially modulates immune-inflammatory crosstalk in IFA-associated nephrotoxicity. This represents one of the novel aspects of present study, as this axis has been discussed only to a limited extent in the context of IFA in the literature.

A shift in the Bax/Bcl-2 balance favoring Bax, coupled with an increase in Caspase-3, reflects the initiation of intrinsic apoptosis via mitochondrial outer membrane permeabilization [84, 85]. IFA induced an increase in Caspase-3 and Bax while suppressing Bcl-2. RA co-treatment counteracted this, limiting apoptosis by reducing Caspase-3 and Bax expression and significantly restoring Bcl-2 levels. Data highlighting the central role of apoptosis in models of IFA-associated Fanconi syndrome are available and the attenuation of apoptosis by natural compounds has been previously reported in IFA toxicity [86, 87–88]. This protective profile is thus confirmed to contribute to the maintenance of tubular epithelial and podocyte integrity in the renal tissue, a finding histologically corroborated at the tissue level by the corresponding Nephrin and Caspase-3 IHC results.

Ferroptosis is a regulated, iron-dependent form of cell death driven by the overwhelming accumulation of lipid hydroperoxides, distinct from classical apoptosis [88, 89]. The critical regulator of this pathway is GPX4, which detoxifies these phospholipid hydroperoxides, while TfR1 upregulation enhances iron influx, fueling lipid-ROS accumulation and strengthening the ferroptotic tendency [90, 91]. In the IFA group, the severe suppression of GPX4 and the upregulation of TfR1 strongly suggest the contribution of this iron-dependent lipid peroxidation to the pathogenesis. This ferroptotic response was further substantiated by the upregulation of PTGS2 (COX-2), a common downstream effector of the pathway. RA co-treatment rebalanced this entire axis, increasing GPX4, decreasing TfR1, and significantly attenuating PTGS2. This pattern implies that RA targets the iron-lipid peroxidation axis, moving beyond simple redox correction. While the structural and molecular protective effects of RA in nephrotoxic models have been previously reported [92, 93], the comprehensive GPX4-TfR1-PTGS2 restoration demonstrated here adds the dimension of ferroptosis modulation to this protective profile.

Histopathology sections provide a direct assessment of the damage to glomerular and tubular architecture; semi-quantitative scoring allows for the objectification of lesion severity [94–96]. IFA produced a distinct tissue phenotype with multiple lesions, such as atrophic glomeruli, dilation of Bowman’s space, widespread tubular dilation, epithelial/podocyte vacuolization, interstitial inflammatory infiltration, and vascular congestion. RA significantly attenuated these patterns, with only limited dilation and mild vacuolization remaining. This morphological improvement ran in parallel with the functional (urea/creatinine) and molecular (redox, inflammation, cell death) corrections, demonstrating that RA’s multi-target effect was reflected in the tissue architecture. KIM-1 is a sensitive indicator of proximal tubule damage [97], while IHC-Caspase-3 reflects apoptosis [98], and Nephrin reflects podocyte/slit diaphragm integrity [99, 100]. The significant increase of KIM-1 in the IFA group and its reduction by RA demonstrates that the tubular epithelium was protected by RA. Similarly, the widespread and intense Caspase-3 staining in the IFA group provides the definitive protein-level validation for the transcriptional (mRNA) upregulation of the intrinsic apoptotic pathway (Bax/Bcl-2/Caspase-3) observed in our molecular analysis. The weakening of Nephrin expression by IFA and its recovery with RA suggests a re-integration of the glomerular filtration barrier (podocyte diaphragm). This triple profile reveals that RA provides simultaneous structural protection on the tubule-podocyte axis, thereby supporting the morphological basis for the improvement in functional parameters.

Taken together, the findings indicate that IFA triggers oxidative stress via CAA-mediated mitochondrial suppression. This oxidative stress, in turn, simultaneously activates ferroptosis (GPX4 ; TfR1) on one hand, and intrinsic apoptosis (Bax/Bcl-2 imbalance; Caspase-3) on the other. Concurrently, it feeds the canonical NF-κB axis via the IL-17 A/ACT1/TRAF6 junction, converting the inflammation into a self-sustaining cycle. RA modulated the critical nodes of this network simultaneously: it restored redox balance, broke the IL-17 A-TRAF6-NF-κB crosstalk, and restrained both the ferroptotic and apoptotic programs. Consequently, RA markedly corrected the tissue architecture and functional outputs. This holistic effect positions RA as a rational adjuvant candidate against IFA-associated nephrotoxicity. Future studies, including protein/nuclear translocation validations (e.g., NF-κB p65, GPX4/TfR1), direct indicators of ferroptosis, and investigations into the efficacy-safety balance in tumor-bearing models, will strengthen the translational step.

## Conclusion

In conclusion, this study confirms that RA, a major polyphenol in common dietary herbs, provides robust nephroprotection against IFA-induced, CAA-mediated toxicity. The core protective mechanism of RA was elucidated, revealing that this functional food ingredient strategically defuses the pathogenic cascade orchestrated by IFA-a cascade involving mitochondrial suppression, severe oxidative stress, intrinsic apoptosis, and ferroptosis. Crucially, RA breaks the self-sustaining inflammation–ferroptosis coupling by disrupting the IL-17 A-TRAF6-NF-κB crosstalk. By restoring redox homeostasis, restraining cell death programs, preserving tissue architecture (both tubular and glomerular), and restoring renal function, these findings position RA as a highly promising nutraceutical adjuvant for mitigating the dose-limiting nephrotoxicity of IFA therapy.

## Data Availability

The datasets generated and analyzed during the current study are available from the corresponding author on reasonable request.
